# Learnable Leakage and Onset-Spiking Self-Attention in SNNs with Local Error Signals

**DOI:** 10.3390/s23249781

**Published:** 2023-12-12

**Authors:** Cong Shi, Li Wang, Haoran Gao, Min Tian

**Affiliations:** 1 School of Microelectronics and Communication Engineering, Chongqing University, Chongqing 400044, China; shicong@cqu.edu.cn (C.S.); wangli@stu.cqu.edu.cn (L.W.); gaohaoran@cqu.edu.cn (H.G.); 2 Key Laboratory of Dependable Service Computing in Cyber Physical Society, Ministry of Education, Chongqing University, Chongqing 400044, China

**Keywords:** spiking convolutional neural networks, learnable leakage coefficient, batch normalization, self-attention, local error signals

## Abstract

Spiking neural networks (SNNs) have garnered significant attention due to their computational patterns resembling biological neural networks. However, when it comes to deep SNNs, how to focus on critical information effectively and achieve a balanced feature transformation both temporally and spatially becomes a critical challenge. To address these challenges, our research is centered around two aspects: structure and strategy. Structurally, we optimize the leaky integrate-and-fire (LIF) neuron to enable the leakage coefficient to be learnable, thus making it better suited for contemporary applications. Furthermore, the self-attention mechanism is introduced at the initial time step to ensure improved focus and processing. Strategically, we propose a new normalization method anchored on the learnable leakage coefficient (LLC) and introduce a local loss signal strategy to enhance the SNN’s training efficiency and adaptability. The effectiveness and performance of our proposed methods are validated on the MNIST, FashionMNIST, and CIFAR-10 datasets. Experimental results show that our model presents a superior, high-accuracy performance in just eight time steps. In summary, our research provides fresh insights into the structure and strategy of SNNs, paving the way for their efficient and robust application in practical scenarios.

## 1. Introduction

Throughout the history of neural network research, traditional artificial neural networks (ANNs) [1] have been the primary focus, due to their remarkable performance and extensive applications. However, despite ANNs’ ability to handle complex nonlinear patterns, a significant gap remains in imitating the functioning of the human brain. Notably, biological neural systems use temporal spike activity, in contrast to ANNs, which heavily rely on continuous activation values. The observation of the human brain’s impressive efficiency in information processing, coupled with its low energy consumption, has sparked interest in spiking neural networks (SNNs) [2].

SNNs differ from ANNs in that they use sparse temporal spike events to encode and process information. While sparse coding does not guarantee an increase in computational power [3,4,5], it does contribute to a reduction in computational complexity, resulting in resource savings. This gain in efficiency gives SNNs a broad potential for application in various fields. In particular, in the context of processing highly dynamic and real-time data streams, SNNs demonstrate superior efficiency in handling temporally correlated information due to their inherent temporal coding properties.

Additionally, the inherent energy efficiency of SNNs offers promising opportunities in areas such as neuromorphic hardware and edge computing. Acknowledging these advantages, the academic community is gradually shifting from ANNs to exploring SNNs, striving for a more genuine representation of biological neural processes, and unlocking novel possibilities in various application areas [6].

In order to facilitate effective training of SNNs, researchers have proposed various methods [7,8,9]. Present research predominantly concentrates on three major aspects: pretraining through clustering and autoencoding, among other methods, under unsupervised learning; enhancing performance by combining supervised information and unlabeled data under semisupervised learning; and implementing backpropagation under supervised learning using alternative differentiable activation functions or other techniques. These three categories of approaches have distinct advantages and disadvantages, but all have exhibited the potential of SNNs in handling signals and various tasks. With the continued advancement of theories and algorithms, SNNs offer a wide range of potential applications. It is worth noting that advanced mathematical theories have also supplied important mathematical tools for the accurate modeling and intricate dynamic analysis of SNNs [10]. 

Unsupervised learning has the ability to adjust neuronal connection weights autonomously by using local information, such as the spike-timing-dependent plasticity (STDP) rule, which modifies weights based on spike-time correlations [11]. These techniques are computationally efficient and simple. However, due to the absence of supervisory information, they generally demonstrate lower accuracy and are often used for network initialization. Semisupervised learning entails pretraining the network using unlabeled data, then fine-tuning it with labeled data. This technique can boost performance by leveraging unlabeled data, but it mandates cleverly designed approaches for utilizing supervisory information [12,13,14]. Currently, supervised learning is the most widespread method for training spiking neural networks. Traditional neural networks use differentiable activation functions and, therefore, they can use the chain rule to calculate gradients directly, enabling backpropagation. On the other hand, spiking neural networks mimic the biologically inspired spike propagation mechanism, making their activation functions nondifferentiable. This makes the direct application of the backpropagation algorithm impossible, posing challenges to the supervised training of spiking neural networks.

Researchers have proposed a variety of supervised learning rules to address issues arising from the nondifferentiability of the spike function in spiking neural networks. For example, SpikeProp [15] employs a linear approximation method, while the alternative gradient rule substitutes traditional activation functions with alternative ones [16]. Moreover, methods based on backpropagation through time (BPTT), which compute gradients jointly from spatiotemporal dimensions, have also gained popularity in recent years [17,18,19,20,21,22]. While these methods have achieved commendable classification accuracy, they come with a substantial computational cost.

In this study, we focus on optimizing both the performance and interpretability of SNNs. Specifically, we improve the conventional LIF model and introduce the LLC-LIF model, where “LLC” stands for “learnable leakage coefficient”. In addition, we propose the batch normalization method combined with the learnable leakage coefficient, termed LLC-BN. By integrating local loss signals and the self-attention mechanism [23] from deep learning, we further enhance the performance and application scope of SNNs. Our primary contributions are as follows:

- We present the LLC-LIF model with a learnable leakage coefficient, which allows the leakage coefficient of the membrane potential to be a learnable parameter. This design provides automated optimization capabilities, ensuring consistent properties between neurons within the same layer and independent properties across layers.
- To better exploit the temporal sensitivity and efficiency of SNNs, we incorporate the self-attention mechanism at the initial time step of the SNN. By integrating strategies from both neuroscience and deep learning, we further enhance the network’s ability to transform temporal and spatial features.
- To adapt to the unique characteristics of spiking neural networks, we introduce the batch normalization method combined with a learnable leakage coefficient, termed LLC-BN. This method harmonizes the temporal dynamics of SNNs with spike-time encoding and enhances the stability and flexibility of the network through joint optimization.
- To efficiently emulate biological neural networks, we introduce local loss signals within the spiking neural network, allowing certain layers to receive distinct learning feedback independently. Using supervised local learning strategies and auxiliary classifiers, we design a hierarchical loss function that ensures excellent performance of the SNN in various tasks.

The rest of this article is organized as follows: Section 2 delves into foundational works relevant to our study. Section 3 details our materials and methods, highlighting our innovative structures and strategies. Section 4 is dedicated to experimental results and comparative analyses. Section 5 concludes our research.

## 2. Related Work

### 2.1. Basic LIF Neurons in SNNs

Neurons, the basic units of the nervous system, consist of a cell body, dendrites, axons, and synapses. The axon’s initial segment serves as the central component, integrating potential changes across the membrane and determining the generation of neuronal spikes. Dendrites process information from other neurons, while axons transmit this information to subsequent neurons. Although traditional ANNs provide a robust computational model, their continuous activation mechanism differs from the spiking response observed in biological neurons. To mimic this biological behavior more accurately, researchers have introduced SNNs. Within the SNN framework, the most representative and widely used neuronal model is the leaky integrate-and-fire (LIF) model. Although the LIF model is only a simplified approximation of real neuronal dynamics, and may not capture all complex neuronal dynamics [24,25], its high computational efficiency in simulating basic electrophysiological properties and spike-response behavior makes it particularly suitable for large-scale neural network simulations. Therefore, despite its limitations, the LIF model remains a valuable choice in SNN applications where a balance between biological realism and computational feasibility is sought.

The LIF neuron in SNNs, inspired by biological paradigms, aims to mimic the intrinsic electrical activities and response behavior of biological neurons [26,27]. Key features of the LIF model include the integration of the membrane voltage potential, its inherent leaky nature, and the firing mechanism that activates when a certain threshold is reached.

At each time step, the LIF neuron accumulates input currents from previous neurons and changes its membrane potential to represent its active state. When the membrane potential accumulates to a certain threshold, the neuron emits a spike, simulating the firing activities observed in biological counterparts. This time-controlled behavior not only brings SNNs closer to the authentic operations of biological neural systems, but also makes them more adept than ANNs at handling time-based information and event-driven tasks.

In the absence of incoming currents, the membrane potential gradually decays due to its intrinsic leakiness. This leak effect is typically defined by a temporal constant, which represents neuronal properties and ensures the biorealism of the model.

The differential equation for the LIF model is as follows:

(1) $$\tau_{m}\frac{dV(t)}{dt}=-(V(t)-V_{rest})+\sum_{i=1}^{n}w_{i}s_{i}(t),$$

where *V*(*t*) represents the membrane potential of the neuron, which varies over time. The term *τ_m_* denotes the membrane time constant, which provides a measure of how swiftly the membrane potential changes. *V_rest_* is the resting potential of the neuron. Each *w_i_* is a synaptic weight, signifying the influence of an incoming synapse from a preceding neuron. *s_i_*(*t*) captures the input spike from the *i* presynaptic neuron, typically represented as 0 or 1, with 1 indicating a spike at time *t* and 0 suggesting the absence of a spike.

The essence of the LIF neuron model lies in simulating the threshold activation features inherent to biological neurons. When the membrane potential of an LIF neuron accumulates to the threshold voltage, *V_th_*, it emits a spike signal, emulating the repolarization phase of the spikes. Concurrently, the membrane potential is reset to the resting potential, emulating the absolute refractory period of biological neurons. As a result, the neuron does not fire consecutively multiple times within a single time step. 

This model provides a concise and intuitive representation of the elementary behavioral attributes of a neuron, positioning it as the preferred choice for the construction of spiking neural networks. Due to its computational simplicity and speed, the LIF model has been widely used in neuroscience education, theoretical investigations, and practical applications. Furthermore, the adaptability of the LIF model’s parameters allows it to replicate a wide range of neuron types, providing a central tool for in-depth exploration and understanding of the electrophysiological properties of neurons.

Within the SNN framework, the LIF model stands as a crucial neuronal model, extensively adopted for its close simulation of neurons’ electrophysiological characteristics. However, current SNN learning methodologies often prioritize synaptic weight adjustments, while less attention is given to critical intrinsic neuronal parameters such as the leakage coefficient. This oversight may neglect the heterogeneity amongst neurons and the significant role of the leakage coefficient in dynamic neuronal behavior, as highlighted in biological studies [28,29]. Addressing this, our research introduces an innovative training approach, which not only targets synaptic weight optimization but also focuses on adaptively fine-tuning the leakage coefficient for individual neurons. This strategy aims to fully exploit the potential of SNNs, enhancing their expressiveness and functional depth to mirror the complex dynamics observed in biological neural systems more effectively.

### 2.2. Self-Attention

After achieving significant success in the field of natural language processing (NLP) [30], the self-attention mechanism has become a crucial research direction in deep learning. Traditional neural network training methods, despite their excellent performance on many tasks, often face significant challenges when dealing with problems involving complex structures and large-scale data, especially in scenarios involving long-range dependencies and intricate interactions. The introduction of the self-attention mechanism can alleviate these limitations. Firstly, self-attention can capture long-range dependencies within the input data, enabling a more comprehensive understanding of data structures and patterns. This is crucial for analyzing intrinsic relationships and interactions between features. Secondly, self-attention can provide a higher-level feature representation that helps the network to better abstract and generalize data, thereby improving the performance and generalization capabilities of the network. Finally, self-attention allows the network to interact between different positions in the input data, which is crucial for tasks involving sequence data, images, and speech, among others.

Through learned parameter transformations, this mechanism computes the internal structure of the input matrix *X* to generate the output matrix *H*. Each element of *H* is a weighted sum of columns from *X*, with the weights determined by the keys, values, and queries—three sets of learnable parameters.

(2) $$Q=W_{q}\cdot X,\;K=W_{k}\cdot X,\;V=W_{v}\cdot X.$$

(3) $$H=\mathrm{Attention}(Q,K,V)=\mathrm{softmax}(K^{T}Q)\cdot V.$$

**Figure 1:**
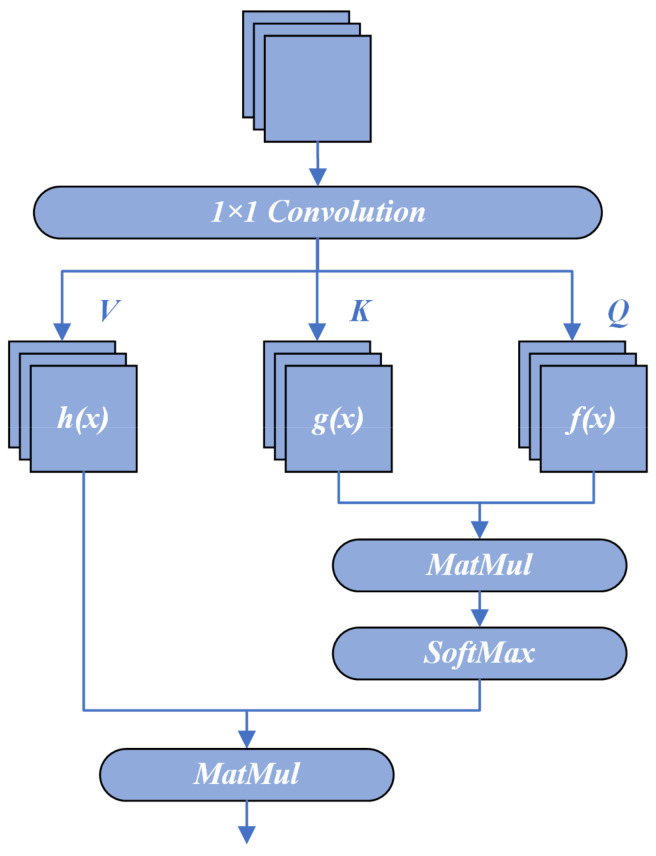
Schematic of the self-attention mechanism.

Inspired by the successes in NLP, researchers have begun to explore the potential of the self-attention mechanism for computer vision tasks. Given the special nature of image data, adaptations to the original mechanism are often necessary. One common approach is to apply three 1 × 1 convolutional kernels to the feature map *X*, producing *f*(*x*), *g*(*x*), and *h*(*x*). These resulting feature maps are then used as the keys, queries, and values in the self-attention process.

(4) $$O=\mathrm{Softmax}(f(x)\cdot g{\left(x\right)}^{T})\cdot h(x).$$

where the weights are computed from the feature maps *f*(*x*) and *g*(*x*) and are used to weight different locations of the feature map *h*(*x*) to obtain the output feature map *O*, as shown in Figure 1. Compared to the original linear transformation method, this approach provides a more intuitive and reasonable way to compute attention weights.

From a theoretical perspective, the self-attention mechanism offers a means to explicitly model dependencies among input features, thereby paving new avenues for semantic understanding and representational learning. In practical applications, the self-attention mechanism dynamically adjusts weights across different regions, enabling the model to focus more precisely on critical portions of the input image. Consequently, this results in significantly sharper and more accurate predictions for a diverse set of computer vision challenges.

The self-attention mechanism has demonstrated its versatility and outstanding performance across various domains. While it has achieved notable success in the NLP sector, it has also spurred a host of innovative applications and theoretical advancements in the field of computer vision. The potential of this technology in multimodal learning, transfer learning, and other areas beckons further exploration and utilization.

Given the exemplary performance of self-attention in conventional deep learning models, especially its proficiency in capturing long-range dependencies and constructing rich contextual relationships [31,32,33,34], there is a growing interest in how it might be applied to other neural network architectures like the spiking neural network. Unlike traditional neural networks, SNNs operate based on temporal dynamics, where information propagates in the form of spikes, emphasizing the model’s role in capturing features and dependencies in the temporal dimension. Therefore, integrating self-attention into SNNs is expected to enhance their capability in processing temporal sequence data and in discerning long-term temporal dependencies more accurately.

### 2.3. Normalization

In deep neural networks and SNNs, vanishing and exploding gradients present pervasive challenges during training, adversely affecting the network’s training stability and convergence rate. 

The distinctive temporal–spatial information processing mechanism in SNNs, coupled with their nondifferentiable spiking activation functions, engenders gradients that are volatile in both temporal and spatial dimensions. Such instabilities impede the efficient backpropagation of errors, making the network arduous to train. Another concern is the activation rate. Throughout the training process, the distribution state of inputs from the preceding layers constantly drifts, leading to anomalous activation rates in subsequent layers and compromising the network’s expressiveness.

To address these issues, introducing normalization in SNNs becomes imperative. Normalization has been demonstrated to bolster the stability of the network training process, ameliorating challenges like vanishing/exploding gradients. Furthermore, normalization layers can recalibrate data distributions, expedite the training pace, and facilitate smoother gradient propagation. Additionally, normalization can dynamically adjust the thresholds and input proportions of individual neurons, harmonizing the overall network activation rate distribution and ensuring the network’s temporal encoding capability.

To address the vanishing and exploding gradient issues, researchers have proposed a number of normalization methods to ensure that gradients remain within a favorable range, thereby promoting stable network training and accelerated convergence [35,36,37,38]. 

However, due to the unique operating mechanism of SNNs, one cannot directly adopt normalization methods from conventional neural networks. It is essential to develop tailored normalization techniques that take into account the specific spatiotemporal information processing and spiking activation characteristics of SNNs. 

### 2.4. Spatiotemporal Backpropagation

SNNs differ markedly from traditional DNNs in terms of forward data flow processing. SNNs not only propagate data hierarchically in the spatial domain but also achieve sustained integration in the temporal domain through each neuron’s self-feedback mechanism, emulating the spatiotemporal behavior of biological neurons. This capability allows SNNs to operate with complex spatiotemporal patterns and encode information through distinct spike patterns.

Given the unique features of SNNs, various modern studies have concentrated on utilizing spatiotemporal domain characteristics in enhancing the performance and learning capacity of SNNs. The emission of spikes by the neuron is determined by the decay of the membrane potential, which is influenced by the input from the neuron’s presynaptic connections. The state of each neuron is shaped collectively by the spatial input received and its time-based memory.

While the conventional backpropagation algorithm has been established to be effective for DNNs, its direct implementation in SNNs is problematic due to the inherent disparities in spatiotemporal dynamics between SNNs and DNNs. In view of this, a spatiotemporal backpropagation algorithm has been proposed [10], specifically designed to train high-performance SNNs. This algorithm offers a more comprehensive integration of the spatial and temporal domains within the network than previous approaches.

**Figure 2:**
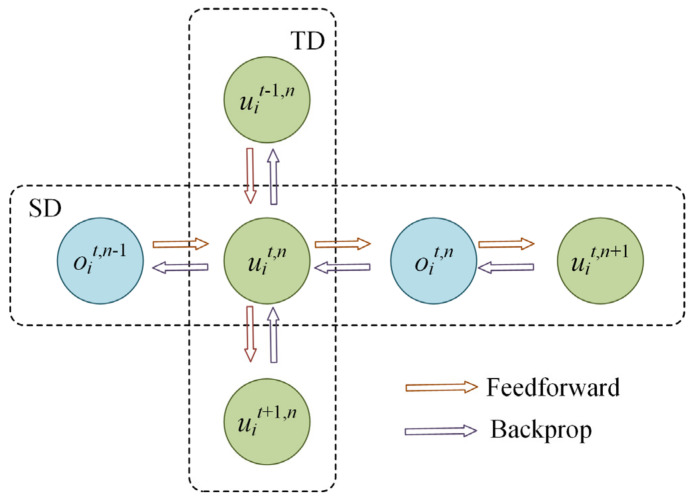
Schematic of spatiotemporal backpropagation. The diagram illustrates the interaction between the spatial domain (SD) and the temporal domain (TD) through feedforward and backpropagation processes.

As shown in Figure 2, the spatiotemporal backpropagation operates in the spatial dimension by relying on the gradient descent method to adjust the network’s weights and thresholds, with the objective of minimizing the error between the network’s output and the desired output. In the temporal dimension, it iteratively updates the neuron’s membrane potential, simulating the neuron’s dynamic response after receiving a spike. This spatiotemporal iterative process not only provides a deeper understanding of how data are transmitted in SNNs but also significantly enhances the network’s learning capability.

In a later study, Wu et al. [39] established the compatibility of the STBP algorithm with the PyTorch framework by transforming the LIF model into an explicit iterative version. As a result of this conversion, training deeper SNNs at a faster rate is possible. The calculation of the cell’s membrane potential 𝑢 is depicted as:

(5) $$\begin{array}{l} u^{t+1,n+1}(i)=k_{\tau }u^{t,n+1}(i)(1-o^{t,n+1}(i))+\sum_{j=1}^{l(n)}w_{ij}^{n}o^{t+1,n}(j),\\ o^{t+1,n+1}(i)=f(u^{t+1,n+1}(i)-V_{th}), \end{array}$$

where *u* and *o* denote the membrane potential and output of the neuron, respectively, *t* represents the current time point of the neuron, *V_th_* represents the membrane potential threshold of the neuron, and *n* and *l*(*n*) represent the *n*-th layer of the network and the number of neurons contained in that layer, respectively. *w_ij_* is the synaptic weight between the *j*-th neuron in the *n*-th layer and the *i*-th neuron in the *n* + 1 layer, and *k_τ_* is a hyperparameter that denotes the rate at which the neuron’s membrane potential decays over time. *f*(*x*) is a step function, and *f*(*x*) = 0 when *x* < 0, otherwise *f*(*x*) = 1. *o^t^*^,*n*+1^ and *o^t^*^+1,*n*^ will jointly affect *o^t^*^+1,*n*+1^ through the update-trigger-reset mechanism.

However, as neural networks grow in complexity, conventional training methods utilizing global loss functions and the backpropagation algorithm face escalating computational and memory challenges. Notably, backpropagation requires the entirety of the forward computation to be completed before initiating weight adjustments, leading to potential inefficiencies. Concurrently, the need to retain comprehensive activation data for backpropagation results in significant memory overheads. Furthermore, the artificial training method employing global error backpropagation does not account for synaptic learning mechanisms in biology that rely on local information. In response to these challenges, a new paradigm is emerging. It leans towards layer-by-layer training methodologies that leverage local loss functions and local classifiers. These methodologies offer improved computational parallelism and efficient memory allocation. They also align better with biologically credible learning mechanisms, which has the potential to bridge the gap between artificial and biological neural systems [40,41,42]. The incorporation of hierarchical supervisory signals, inherent to this approach, can enhance the network’s representational capabilities. In conclusion, implementing local loss functions and classifiers for training presents multiple benefits, establishing it as a crucial direction for future neural network training methodologies. This study delves deeper into this premise, aiming to enhance and adapt these methods for practical system training.

## 3. Materials and Methods

### 3.1. LIF Model with Learnable Leakage Coefficient (LLC-LIF)

In neuronal dynamics, the leakage coefficient of the membrane potential is a crucial factor, defining the velocity at which a neuron’s membrane potential goes back to its resting state when there are no external inputs present. When examining the biological plausibility of the LIF model, we stress the importance of the leakage coefficient in the model. This factor is key to the simulation of the organic degeneration of the membrane potential of neurons to the resting state. This process is mainly influenced by ion channels, with potassium channels playing a critical role in maintaining and restoring the resting potential [43].

The LIF model adjusts the leakage coefficient to simulate the dynamics of the membrane potential in neurons, taking into account ion channel availability and conductance changes. By decreasing the leakage coefficient, the neuron’s integration time for inputs can be extended, resembling neurons with closed ion channels and altering their firing patterns [44].

The rate of leakage is intricately associated with the membrane time constant *τ*, which signifies the duration over which a neuron combines input information. The magnitude of the leakage coefficient directly affects how neurons respond to temporal patterns of input and their encoding abilities. Hence, it is imperative to regulate the leakage coefficient to mirror the biological traits of real neurons while creating neuronal models.

Diverging from traditional LIF models that employ a fixed leakage coefficient, we introduce a novel LIF model. In this proposed model, the membrane potential’s leakage coefficient is devised as a learnable parameter, termed LLC-LIF. The dynamical equations governing this neuron model are as follows:

(6) $$\begin{array}{l} u^{t+1,n+1}(i)=k_{\tau }(a)u^{t,n+1}(i)(1-o^{t,n+1}(i))+\sum_{j=1}^{l(n)}w_{ij}^{n}o^{t+1,n}(j),\\ o^{t+1,n+1}(i)=f(u^{t+1,n+1}(i)-V_{th}), \end{array}$$

where *k_τ_*(*a*) represents a clamping function bounded between (0, 1), ensuring that *τ* = 1/*k_τ_*(*a*) lies within the range (1, +∞). In our experiments, we set *k_τ_*(*a*) = 1/(1 + exp(−*a*)).

The learnable leakage coefficient, denoted as *k_τ_*(*a*), offers several biologically plausible advantages. Its automatic optimization obviates the need for manual hyperparameter selection, facilitating end-to-end and automated model training. Neurons within the same layer share the value of *k_τ_*(*a*) and exhibit similar characteristics. However, according to Eve Marder et al. [45], there is a certain degree of individual variability among these neurons. In our future work, we may explore how such individual differences impact the network’s training performance, presenting an interesting and valuable research direction. Moreover, leakage coefficients are independent across different layers, endowing neurons in each layer with unique temporal coding properties. 

By integrating this adjustable leakiness into the model, we can more accurately imitate the dynamic behavior of biological neurons. As this parameter is trainable, it can adaptively modify during the training process based on data. This flexibility can facilitate the model in acquiring the optimum leaky behavior, thereby maximizing its performance on particular tasks. This biologically rooted design creates opportunities for constructing SNNs. Our research aims to utilize the LLC-LIF model in order to train efficient SNNs to tackle complex temporal learning challenges.

### 3.2. Onset-Spiking Self-Attention (OSSA)

SNNs provide a unique and energy-efficient framework for neural computation by emulating the spiking propagation mechanism of biological neurons. In SNNs, time plays a pivotal role, particularly when using spatiotemporal backpropagation for effective training. However, despite the irreplaceable importance of time in SNNs, traditional training strategies still have limitations when it comes to handling temporal information and feature selection. To harness the full potential of SNNs, we propose introducing a self-attention mechanism at the initial time step.

Biological research has revealed that the brain’s initial response after receiving a stimulus is crucial in subsequent information processing and decision-making processes [46]. This initial response provides critical information about the stimulus and may form the basis for information processing and decision-making. In the context of SNNs, this suggests that the initial time step *T* = 0 might also play a decisive role in the entire network’s response. By enhancing feature selection at this crucial moment, we aim to enable the network to focus more on genuinely important information in subsequent time steps.

In this context, the self-attention mechanism provides a promising approach. Initially introduced in the transformer model, it allows the model to assign different weights to each element in the input sequence, capturing long-range dependencies within the sequence. By assigning weights to each input element, this mechanism enables the model to better focus on the most crucial parts of the entire sequence. When we apply this mechanism to SNNs, we hope that the network can better identify and respond to key time points and features, leading to more accurate responses throughout the time sequence.

Considering these factors, we believe that introducing self-attention into the initial stage of SNNs is a reasonable and promising approach. This new method combines successful strategies from deep learning with insights from neuroscience, offering a new direction for further research and application of SNNs.

Traditional implementations of self-attention typically use a simple convolutional layer to transform input features. However, when considering SNNs, the network’s dynamic nature and spiking behavior provide an opportunity to further optimize the attention mechanism. To achieve this, we propose changing the convolutional layer of self-attention from a simple two-dimensional convolutional layer to one integrated with optimized LIF structures. The core idea behind this change is to leverage the dynamic properties of the optimized LIF structure to enhance the representational capacity of the attention mechanism.

We consider incorporating the influence of spiking neurons after computing queries, keys, and values, and before calculating the weight coefficients, as shown below:

(7) $$\begin{array}{l} f^{\prime }(x)=LLC\_LIF(f(x)),\;\\ g^{\prime }(x)=LLC\_LIF(g(x)),\;\\ h^{\prime }(x)=LLC\_LIF(h(x)). \end{array}$$

Then we can use *f*′(*x*), *g*′(*x*) and *h*′(*x*) to compute the weight coefficients and generate the output of self-attention.

(8) $$O’=\mathrm{Softmax}(f^{\prime }(x)\times g^{\prime }{\left(x\right)}^{T})\times h^{\prime }(x).$$

By combining the two-dimensional convolution layer with the optimized LIF structure, we can balance feature transformations in both spatial and temporal dimensions. While traditional two-dimensional convolution layers focus solely on spatial information, the optimized LIF structure provides a means of temporal modulation, allowing the model to adaptively handle dependencies at different time scales.

In summary, modifying the convolution layer of self-attention to incorporate the optimized LIF structure not only enhances the model’s representational capacity but also offers a means of adaptive temporal modulation. This is crucial for pulse neural networks. Experimental results have demonstrated significant performance improvements on various benchmark tasks with the introduction of pulse-based self-attention, further validating the effectiveness and potential of our approach.

### 3.3. Learnable Leakage Coefficient Batch Normalization (LLC-BN)

Traditional batch normalization techniques have shown significant efficacy when applied to conventional neural networks, but they are not directly adaptable to SNNs. This incompatibility arises due to the temporal dynamics of membrane potentials in SNNs and the unique time-encoding characteristics of spikes, which are fundamentally different from networks with static activation functions.

To address this, we propose a novel normalization technique tailored for the operational mechanism of SNNs: the learnable leakage coefficient batch normalization (LLC-BN) method. This method jointly optimizes the neuron’s membrane potential leakage coefficient and input normalization. It computes the mean and variance of the membrane potential at each time step as normalization benchmarks, smoothing the temporal activation patterns of the network. This advanced normalization approach takes into account the spatiotemporal information representation traits of SNNs. By co-optimizing the leakage parameter and the input distribution, it effectively reduces the variance of temporal encoding, enhancing the network’s capability to learn dynamic features.

In SNNs, each neuron’s behavior is time-based, responding in the form of spikes across different time steps. Let *o_t_* denote the spike outputs of all neurons in a layer at time step *t*. To characterize how neurons respond to their inputs, we introduce a convolutional kernel *W* and bias *B*. For a given input *x_t_*, its spike response is transformed through the convolutional kernel *W* and bias *B*. This can be mathematically represented as:

(9) $$o_{t}=f(W_{K}*x_{t}+B),$$

where * denotes the convolution operation, and *f* serves as an activation function. Typically, in spiking neural networks, *f* acts as a threshold function, deciding whether to fire a spike. *x_t_* is a four-dimensional tensor representing the presynaptic input at time step *t*. N stands for the batch size, indicating the number of samples processed simultaneously. *C* refers to the number of channels, representing the count of input features. *H* and *W*, on the other hand, represent the height and width of the input, symbolizing the spatial dimensions.

In the proposed LLC-BN method, normalization is performed along the channel dimension. Specifically, for each channel feature map *x_k_*, it undergoes the following normalization process:

(10) $$x_{k}{}^{\prime }=\frac{\alpha k_{\tau }(a)(x_{k}-E[x_{k}])}{\sqrt{Var[x_{k}]+\epsilon }},$$

subsequently, the normalized output obtained is represented as:

(11) $$y_{k}=\lambda_{k}x_{k}{}^{\prime }+\beta_{k},$$

where *α* is a hyperparameter, *ϵ* is a small constant to prevent division by zero, *k_τ_*(*a*) is a trainable leakage parameter, and *x_k_* and *x_k_*′ are the neural inputs before and after normalization, respectively. *λ_k_* and *β_k_* are two trainable parameters used for scaling and shifting in the linear transformation after normalization. *E*[*x_k_*] and *Var*[*x_k_*] denote the mean and variance calculated from the elements of *x_k_* along the batch axis *N*, the spatial axes *H* and *W*, and the time axis *T*. Specifically, *y_k_* denotes the normalized presynaptic input received by the *k*-th channel neuron in the subsequent layer over a period of time *T*.

Moreover, we do not just compute the mean and variance for the current batch of data; we also employ the moving average method to estimate the mean *μ_inf_* and variance *σ_inf_*^2^ over the entire dataset. This strategy ensures robust normalization during the inference phase, irrespective of the batch size of the input data.

Of particular note in LLC-BN, the pre-activation is normalized to a distribution with a mean of 0 and a variance of α^2^ × *k_τ_*(*a*)^2^, differing from the *N*(0,1) in traditional batch normalization. This adjustment makes the normalization more attuned to the spiking behavior of SNNs.

As can be observed, the aforementioned self-optimizing leakage coefficient *k_τ_*(*a*) is introduced to adjust and scale the normalized data. in line with biological systems. The leaky parameter *k_τ_*(*a*) dynamically adjusts the normalization range, enabling flexible adaptation to data distribution and variations. All technical terms are explained when first used. This parameter enhances neural sensitivity to historical information and time sensitivity of normalization. Additionally, *k_τ_*(*a*) ensures stability during normalization, particularly when dealing with noise or outlier data. Moreover, its trainability allows for self-adjustment during training and thus optimizes the model’s performance across various tasks and data distributions. During inference, the standard batch normalization strategy is followed, wherein the moving average over the complete dataset is employed for estimating the mean and variance, thus ensuring the stability of the network.

In order to implement the SNN on neuromorphic hardware whilst preserving its full spiking properties, we adopted the batch normalization scale fusion technique. This approach eliminates the need for batch normalization during the inference stage, enabling the entire network to maintain a pure spiking form and making it simpler to deploy on neuromorphic platforms. Let *W*′ and *B*′ denote the convolutional kernel and bias, respectively, following normalization. After batch normalization scale fusion, these weights and biases undergo corresponding transformations:

(12) $$W^{\prime }=\frac{\lambda \alpha k_{\tau }(a)W}{\sqrt{\sigma_{inf}^{2}+\epsilon }},$$

(13) $$B^{\prime }=\frac{\lambda \alpha k_{\tau }(a)(B-\mu_{inf})}{\sqrt{\sigma_{inf}^{2}+\epsilon }}+\beta .$$

During the inference process, information is passed layer by layer through these transformed weights *W*′ and biases *B*′ without the need for additional batch normalization steps. This means that LLC-BN only affects the computation during training and does not affect the operating mechanism of a trained SNN. In our experiments, we initialise the trainable parameters *λ* and *β* to 1 and 0, respectively. The hyperparameter *α* is set to 3.2.

### 3.4. Spatiotemporal Backpropagation with Local Error Signals

At the core of biological neural networks are synapses, which interact within highly complex and parallel environments, often relying on locally available information to adjust their weights. This phenomenon suggests that SNNs, when simulating biological neural systems, could similarly benefit from the drive of local information, thereby enhancing the training efficiency and accuracy of the network. Understanding this context led us to introduce local loss signals in SNNs. In this setup, each layer can be independently updated based on local learning signals, making the training of SNNs more efficient. This strategy aligns with the parallelism and adaptability observed in biological neural networks and better accommodates the spatiotemporal characteristics of information in SNNs.

**Figure 3:**
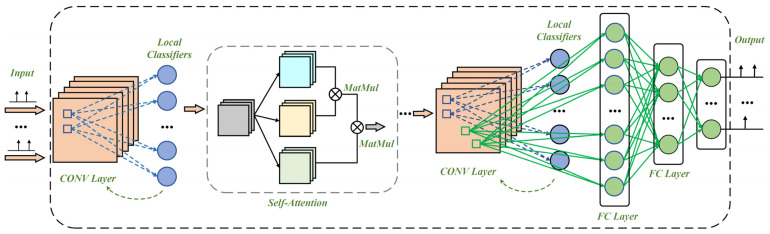
The comprehensive architecture diagram of the network, which incorporates supervised local loss, highlights the synergistic interplay between independent local losses at convolutional layers and the global loss, reflecting the adaptability and spatiotemporal dynamics of biological neural systems.

The complete network structure is shown in Figure 3. Within our network, we have integrated a supervised local learning approach, the core of which is the use of auxiliary classifiers to construct hierarchical loss functions [47]. This allows us to utilize training labels for more explicit and targeted local updates while ensuring that SNNs perform well across a variety of tasks. Furthermore, our local loss signal strategy not only draws inspiration from the local learning mechanisms of biological neural networks but also integrates ideas from deep continuous local learning, leveraging temporal local information for continuous SNN training at each time step.

The primary advantage of this approach lies in providing more direct and specific feedback for hidden layers. Local losses can indicate more explicitly which part of the network needs adjustment, rather than relying on global feedback propagated from the output layer. This enables us to fine-tune each part of the network more precisely, capturing and learning subtle differences in the data more effectively. Additionally, introducing local loss signals brings added training efficiency. Each layer can be updated independently and in parallel, making the training process more efficient and facilitating faster convergence to optimal solutions.

In summary, by introducing local loss signals into spatiotemporal backpropagation, we not only enhance the ability of spiking neural networks to handle complex data patterns, but also greatly improve training efficiency and stability.

In our research, we adopted standard convolutional and fully connected network architectures. One significant feature of this model, compared to traditional neural network structures, is the assignment of independent local losses between convolutional layers. These local losses, combined with a global loss, collectively form the total loss of the network to guide the optimization process.

Specifically, the introduction of local losses aims to ensure that each convolutional layer can independently optimize and capture its corresponding feature space. The global loss, on the other hand, aims to ensure that the macrolevel outputs of the network match the expected labels as closely as possible, thus achieving the overall training goal of the model. To quantify the difference between the model outputs and the real labels, we used the mean squared error (MSE) [48] as the loss function, defined as:

(14) $$L_{\mathrm{MSE}}=\frac{1}{N_{s}}\sum_{i=1}^{N}{\left(y_{i}-{\hat{y}}_{i}\right)}^{2},$$

where *y_i_* represents the true values, *ŷ* represents the model’s predictions, and *N_s_* is the number of samples.

The comprehensive loss function of the model is composed of the local losses from all the convolutional layers and a global loss, and can be expressed as follows:

(15) $$L_{\mathrm{total}}=\sum_{i=1}^{n}L_{\mathrm{locali}}+L_{\mathrm{global}},$$

where *n* represents the total number of convolutional layers, and *L*_locali_ is the local loss for the *i*-th convolutional layer.

## 4. Experiments

### 4.1. Benchmark Datasets

We evaluated our proposed SNN model on three primary image datasets: MNIST [49], FashionMNIST [50], and CIFAR-10 [51]. Specifically, the MNIST dataset consists of 10 classes of handwritten digit images with a resolution of 28 × 28, totaling 50,000 training samples and 10,000 test samples. FashionMNIST, structurally similar to MNIST, showcases 10 different clothing categories. On the other hand, the CIFAR-10 dataset encompasses 10 object classes, each with images of 32 × 32 resolution, comprising 50,000 training images and 10,000 test images. The detailed attributes of these datasets, such as image resolution, number of categories, and the division of training/testing subsets, are all listed in Table 1.

### 4.2. Network Structure Configuration

In the experiments, the network architecture “128c3-p2-128c3-p2-2048-100-10” was employed for the MNIST and FashionMNIST datasets. For the CIFAR-10 dataset, the architecture “256c3-256c3-256c3-p2-256c3-256c3-256c3-p2-2048-100-10” was adopted. In these specifications, “c” represents a convolution layer with the preceding number indicating the quantity of convolution kernels. The number “3” that follows elucidates the kernel size of 3 × 3. The symbol “p” denotes a pooling layer, with the succeeding “2” indicating a 2 × 2 pooling window size. Additionally, “2048” and “100” symbolize fully connected layers with their respective neuron quantities.

At the tail end of the fully connected layers, a unique “100-10” structure was incorporated. Notably, “100-10” does not directly signify two adjacent fully connected layers. In this context, “10” pertains to an average pooling layer applied to the output of the preceding fully connected layer, with both stride and window size set to 10. The core intention of this strategy is to first project complex features onto a relatively low-dimensional 100-feature space, and subsequently obtain a 10-dimensional output representation via the average pooling layer. This approach accomplishes feature dimension reduction, streamlines the network architecture, and preserves pivotal information while mitigating computational demands. Following this, averaging features in a low-dimensional space enables the model to capture more prominent and significant information, elevating the capability of recognizing key features and, to a degree, suppressing noise.

During the network training phase, dynamic optimization of network parameters was conducted via learnable leakage coefficients and the OSSA strategy. The introduction of the novel normalization method, LLC-BN, also augmented the network’s capability to learn dynamic features. Furthermore, to elevate network performance, the local error signal was employed, propelling the network to achieve more efficient feature extraction and representation across layers, thereby assisting the model in learning the mapping relationship from input to output with increased stability. To enhance the network’s generalization capability and combat overfitting, a dropout strategy [52] was implemented in the latter part of the model. This strategy, by suppressing the activation of random neurons during training, offers robust regularization effects, ensuring a more resilient network and preventing the model from excessively relying on specific neurons in the training data, thus promoting a more robust and sturdy training process.

In this study, the rate coding method [53] was utilized to transform pixel values of images into spikes within the spiking neural network. Rate coding is an encoding strategy where a neuron’s spike firing rate is directly proportional to its input strength. This implies that a higher input strength would result in a greater spike firing rate. A notable advantage of this encoding strategy is its ability to intuitively reflect the strength of input data, furnishing spiking neural networks with ample input information. Using rate coding ensures that SNNs receive temporal spike information directly correlated with the original image pixels, laying a solid foundation for subsequent neural network processing. Additionally, in our model, the output employs a direct decoding strategy, which directly presents the total spike count. For the loss calculation phase, these total spikes are transformed into spike frequencies to facilitate comparison with the target labels, which are in one-hot encoded form.

All experiments were implemented using SpikingJelly [54], an open-source SNN deep learning framework built upon PyTorch [55]. We trained our models on NVIDIA GeForce GTX 3090. For the experiments across the MNIST, FashionMNIST, and CIFAR-10 datasets, we consistently set the batch size to 16 and employed the Adam optimizer [56]. All networks were trained for a total of 200 epochs. Our source code is available at https://github.com/CQU0121WL/Learnable-Leakage-and-Onset-Spiking-Self-Attention-in-SNNs-with-Local-Error-Signals (accessed on 7 December 2023).

### 4.3. Work Comparison and Discussion

For the MNIST dataset, it is evident that various methods have demonstrated excellent performance in the training and optimizing of deep spiking neural networks, as shown in Table 2. However, our approach significantly surpasses all other methods listed by achieving an accuracy of 99.67% with only eight time steps. Jin et al. [57] employed similar network structures and trained over more time steps, resulting in accuracies close to ours but requiring significantly more time steps. This might be attributed to their training strategies, which emphasized spatiotemporal backpropagation and mixed macro/microlevel backpropagation. Sengupta et al. [58] and Lee et al. [21] opted for the LeNet-5 structure, and although their accuracy was comparable to our approach, their network structures and time steps were less efficient than ours. Zhang et al. [59] achieved 99.62% accuracy by introducing recursive layers into the network, but still required a lengthy 400 time steps. Wu et al. [19] and Cheng et al. [60] used a simpler network structure with relatively few time steps, resulting in moderate accuracy. Hu et al. [61] chose a more complex network structure like ResNet-8, demonstrating that direct training of SNNs is possible even in more intricate networks. Notably, Fang et al.’s approach [62] is remarkable as it not only trained the network’s weights but also learned membrane time constants, potentially adding more dynamism to SNNs. To ensure a fair comparison, we employed the same batch size and epochs for the method of Fang et al. as our own, attaining an accuracy rate of 99.60%. Ma et al.’s three methods [63], FELL, BELL, and ELL, all emphasized spike learning based on local classifiers, indicating the effectiveness of local learning in deep networks. Gao et al. [64] adopted the VGG-9 structure and employed a quantized training framework for the conversion from deep ANNs to SNNs. While this approach is technically noteworthy, it required significantly more time steps than our method.

In comparative experiments on the FashionMNIST dataset, as shown in Table 3, Cheng et al. [60] proposed LISNN, which incorporates lateral interactions to improve the noise robustness of SNNs and achieved an accuracy of 92.06%. Zhang et al. [59] introduced a spike train level backpropagation method for training deep recurrent spiking neural networks. Despite their excellent performance in spatiotemporal learning and event-driven neuromorphic processors, the accuracy of Zhang et al.’s method [59] was 90.13%. In a different approach, the TSSL-BP method [22] effectively trained deep SNNs in just a few time steps, improving accuracy to 92.73% on various image classification datasets. By the 200th epoch, Fang et al.’s algorithm [62] had achieved an accuracy of 93.67%. In addition, other research methods such as FELL, BELL and ELL [63] achieved accuracies of 92.91%, 92.90% and 92.51%, respectively. Although these techniques share architectural similarities with our proposed approach, they still lag behind in terms of accuracy, highlighting the innovative nature of our method.

Each of these methods has introduced its own innovations and optimization strategies. However, our proposed approach showed superior performance on the FashionMNIST dataset according to the experimental results. These differences can be attributed to differences in learning algorithms, initialization of network parameters, and fine-tuning of network structures. These results further validate the superiority and effectiveness of our method for training deep spiking neural networks.

In comparative experiments on the CIFAR-10 dataset, a variety of methods and architectures were employed, as shown in Table 4. Sengupta et al. [58] utilized the VGG-16 architecture and achieved an accuracy of 91.55%. Han et al. [65] opted for the ResNet-20 and introduced a spiking neuron model with a “soft reset”, recording an accuracy of 91.36%. Kundu et al. [66] reduced spike activity through attention-guided compression, resulting in an accuracy of 89.84%. Most of these methods predominantly relied on the ANN2SNN training paradigm. Rathi et al. [67] employed a hybrid training approach, leveraging converted SNN weights and thresholds as initial values, and achieved an accuracy of 92.02%. DECOLLE [47], while emphasizing continual local learning, attained an accuracy of only 74.70%. Y. Wu et al. [39] underscored the significance of directly training SNNs and achieved an accuracy of 90.53% within 12 time steps. Lee et al. [21] deployed the ResNet-11 architecture and secured an accuracy of 90.95%. A distinctive feature of TSSL-BP [22] was the introduction of a novel temporal learning backpropagation method, successfully reaching an accuracy of 89.22%. Ledinauskas et al. [68], employing the ResNet-11 architecture, achieved an accuracy of 90.20%. Fang et al. [62] highlighted the importance of the membrane time constant in their model, achieving an accuracy of 91.71% with the same batch size and number of epochs as ours. Kim et al. [69] reached an accuracy of 90.50% within just 25 time steps. Utilizing local classifier techniques, FELL, BELL, and ELL [63] reported accuracies of 88.13%, 86.24%, and 84.55%, respectively. In comparison, our model attained a remarkable 92.08% accuracy in a mere eight time steps, demonstrating innovative superiority over other methods and adeptly balancing network depth, time steps, and accuracy.

**Figure 4:**
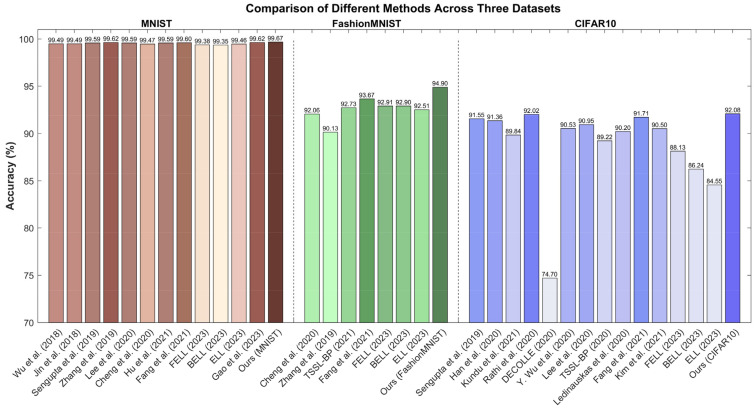
The visual comparison of different methods [19,21,22,39,47,57,58,59,60,61,62,63,64,65,66,67,68,69] cross three datasets. Within each dataset, darker colors of the bar graphs indicate higher accuracy.

The comparative visualization of the results across different datasets for different methods is shown in Figure 4. Overall, these studies indicate that various network architectures, training strategies, and optimization techniques have a significant impact on the performance of SNNs. However, our approach distinctly excels when considering factors like network complexity, required time steps, and accuracy. This superiority can likely be attributed to our unique learning algorithms and optimization techniques.

### 4.4. Ablation Study

To systematically assess the specific contributions of the techniques we introduced on the performance of spiking neural networks, we conducted an ablation study using the FashionMNIST dataset with a batch size of 16 and training for 200 epochs, as shown in Table 5. Initially, we discussed the performance of the model when all the innovative techniques proposed in this paper were applied. The results indicated an accuracy of 94.90%, providing a critical benchmark for our comparisons. Further, by excluding the LLC-LIF while keeping other techniques intact, there was a slight performance drop to 94.58%. This decline underscores the pivotal role of LLC-LIF in optimizing SNN performance. However, when we removed the local error signal and retained all other techniques, performance decreased marginally to 94.68%, illustrating the significance of the local error signal in enhancing the SNN’s performance. A deeper investigation revealed that omitting LLC-BN led to a performance of 94.85%, and without OSSA, the accuracy stood at 94.87%. Both results suggest the respective contributions of LLC-BN and OSSA to SNN performance, albeit not as pronounced as LLC-LIF. Intriguingly, when both LLC-BN and OSSA were removed simultaneously, performance dropped to 94.72%, implying a cumulative effect when these two techniques are jointly applied. Lastly, in the most simplified model using only LLC-LIF, accuracy further waned to 94.47%, not only re-emphasizing the essential role of LLC-LIF but also highlighting the collective impact of other techniques in boosting performance. In conclusion, LLC-LIF and the local error signal are fundamental in enhancing SNN performance, while the synergy of LLC-BN and OSSA with other techniques can also yield substantial performance gains.

## 5. Conclusions

In this study, we proposed a combination of strategies and techniques to optimize the performance of deep SNNs, and the results demonstrate remarkable potential and superior performance in image recognition tasks. Initially, the LIF architecture was refined, particularly by adjusting its leakage coefficient, allowing SNNs to process data more robustly and efficiently. Furthermore, the integration of a self-attention mechanism at the initial time step enabled the SNN to focus on and capture essential information more effectively, thereby ensuring its accuracy in recognition tasks. Additionally, we introduced a novel normalization method called LLC-BN to further enhance the network’s stability. By combining this optimized LIF structure with LLC-BN, we achieved a more balanced feature transformation both temporally and spatially. To enhance the training efficacy of SNNs, the use of the local loss signal strategy significantly improved its training parallelism and adaptability. We evaluated the proposed method for classification tasks on MNIST, FashionMNIST, and CIFAR10 datasets. The experimental results show that the proposed method outperforms the state-of-the-art accuracy with only eight time steps. These findings not only attest to the efficiency and robustness of the proposed strategies but also highlight the immense potential of SNNs in image processing tasks. In conclusion, we propose a novel, efficient, and robust framework for SNNs in image processing. Looking forward, by incorporating more biologically inspired techniques, introducing additional optimization strategies, and considering the broader real-world application scenarios of SNNs, deep spiking neural networks are poised for a vast horizon of research and applications. This suggests their potential to bring about tangible value and transformation in various scenarios.

## Figures and Tables

**Table 1:** Benchmark datasets.

Dataset Name	Image Size	Categories	Training Samples	Testing Samples
MNIST	28 × 28	10	50,000	10,000
FashionMNIST	28 × 28	10	50,000	10,000
CIFAR-10	32 × 32	10	50,000	10,000

**Table 2:** Performance comparison with other methods on the MNIST dataset.

Method	Architecture	Time Steps	Accuracy (%)
Wu et al. (2018) [19]	128c3-p2-128c3-p2-2048-100-10	10	99.49
Jin et al. (2018) [57]	15c5-p2-40c5-p2-300-10	400	99.49
Sengupta et al. (2019) [58]	LeNet-5	2500	99.59
Zhang et al. (2019) [59]	784-400-400r-10 (r represent recurrent layer)	400	99.62
Lee et al. (2020) [21]	LeNet	100	99.59
Cheng et al. (2020) [60]	128c3-p2-128c3-p2-2048-100-10	10	99.47
Hu et al. (2021) [61]	ResNet-8	350	99.59
Fang et al. (2021) [62]	128c3-p2-128c3-p2-2048-100-10	8	99.60
FELL (2023) [63]	128c3-p2-128c3-p2-2048-100-10	10	99.38
BELL (2023) [63]	128c3-p2-128c3-p2-2048-100-10	10	99.35
ELL (2023) [63]	128c3-p2-128c3-p2-2048-100-10	10	99.46
Gao et al. (2023) [64]	VGG-9	128	99.62
Ours	128c3-p2-128c3-p2-2048-100-10	8	99.67

**Table 3:** Performance comparison with other methods on the FashionMNIST dataset.

Method	Architecture	Time Steps	Accuracy (%)
Cheng et al. (2020) [60]	128c3-p2-128c3-p2-2048-100-10	10	92.06
Zhang et al. (2019) [59]	784-400-400r-10	400	90.13
TSSL-BP (2021) [22]	128c3-p2-128c3-p2-2048-100-10	10	92.73
Fang et al. (2021) [62]	128c3-p2-128c3-p2-2048-100-10	8	93.67
FELL (2023) [63]	128c3-p2-128c3-p2-2048-100-10	10	92.91
BELL (2023) [63]	128c3-p2-128c3-p2-2048-100-10	10	92.90
ELL (2023) [63]	128c3-p2-128c3-p2-2048-100-10	10	92.51
Ours	128c3-p2-128c3-p2-2048-100-10	8	94.90

**Table 4:** Performance comparison with other methods on the CIFAR-10 dataset.

Method	Architecture	Time Steps	Accuracy (%)
Sengupta et al. (2019) [58]	VGG-16	2500	91.55
Han et al. (2020) [65]	ResNet-20	2048	91.36
Kundu et al. (2021) [66]	VGG-11	100	89.84
Rathi et al. (2020) [67]	VGG-16	200	92.02
DECOLLE (2020) [47]	VGG-9	10	74.70
Y. Wu et al. (2020) [39]	VGG-8	12	90.53
Lee et al. (2020) [21]	ResNet-11	100	90.95
TSSL-BP (2020) [22]	AlexNet	5	89.22
Ledinauskas et al. (2020) [68]	ResNet-11	20	90.20
Fang et al. (2021) [62]	256c3-256c3-256c3-p2-256c3-256c3-256c3-p2-2048-100-10	8	91.71
Kim et al. (2021) [69]	VGG-9	25	90.50
FELL (2023) [63]	256c3-256c3-256c3-p2-256c3-256c3-256c3-p2-2048-100-10	10	88.13
BELL (2023) [63]	256c3-256c3-256c3-p2-256c3-256c3-256c3-p2-2048-100-10	10	86.24
ELL (2023) [63]	256c3-256c3-256c3-p2-256c3-256c3-256c3-p2-2048-100-10	10	84.55
Ours	256c3-256c3-256c3-p2-256c3-256c3-256c3-p2-2048-100-10	8	92.08

**Table 5:** Results of ablation study indicating the specific contributions of various techniques to SNN performance, where “√” indicates the technique was applied and “×” indicates it was not.

LLC-LIF	Local Error Signal	LLC-BN	OSSA	Accuracy (%)
√	√	√	√	94.90
×	√	√	√	94.58
√	×	√	√	94.68
√	√	×	√	94.85
√	√	√	×	94.87
√	√	×	×	94.72
√	×	×	×	94.47

## Data Availability

Data are contained within the article.
